# Which is the optimal adjuvant chemotherapy for resected pancreatic ductal adenocarcinoma? A protocol for a network meta-analysis of randomized controlled trials

**DOI:** 10.1097/MD.0000000000015761

**Published:** 2019-05-24

**Authors:** Qiancheng Hu, Xin Wang, Ye Chen, Xiaofen Li, Peng Cao, Dan Cao

**Affiliations:** Department of Abdominal Oncology, Cancer Center, West China Hospital, Sichuan University, Chengdu, China.

**Keywords:** adjuvant chemotherapy, network meta-analysis, pancreatic ductal adenocarcinoma

## Abstract

**Background:**

Although adjuvant chemotherapy has been shown to reduce relapse and prolong survival after surgery, it is still unclear which adjuvant chemotherapy regimen will be favorable over the all adjuvant treatments evaluated for patients with resected pancreatic ductal adenocarcinoma.

**Methods:**

PubMed, Embase (Ovid version), Cochrane Library, the American Society of Clinical Oncology, and ClinicalTrials.gov database will be searched from their inception to January 19, 2019. We will include studies that contain adjuvant chemotherapy following surgery in patients with pancreatic ductal adenocarcinoma. The outcomes are overall survival, disease-free survival, and grade 3–4 hematological and nonhematological toxicity. The risk of bias for each randomized controlled trial will be assessed as low, moderate, or high using Cochrane Collaboration's tool independently. Pairwise and network meta-analysis will be performed using STATA 13.0, GeMTC, and WinBUGS, respectively. The competing adjuvant chemotherapy regimens will be ranked by an advantage index.

**Results:**

The study is ongoing and the results will be submitted to a peer-reviewed journal for publication.

**Conclusion:**

This network meta-analysis will systematically provide suggestions to select optimum adjuvant treatment for clinical practice in the future.

PROSPERO registration number: CRD42019123907 (https://www.crd.york.ac.uk/PROSPERO/#searchadvanced).

## 1 Introduction

Pancreatic ductal adenocarcinoma (PDAC) is one of the leading causes of mortality.^[1]^ Despite advances in surgery and adjuvant chemotherapy, the overall *prognosis* for the majority *is dismal*, with 10-year survival rate of <4%.^[2,3]^

A 6-month regimen of monoadjuvant therapy with gemcitabine or fluoropyrimidine was recognized as standard of care for resected PDAC.^[4–8]^ The European Study Group for Pancreatic Cancer-4 trial showed significant improvements in overall survival (OS) with use of gemcitabine combined with capecitabine (GX) as adjuvant chemotherapy vs gemcitabine alone (hazard ratio, HR, 0.82; 95% confidence interval, CI, 0.68 to 0.98; *P* = .032).^[9]^ Recently, adjuvant therapy with a modified Folfirinox regimen (oxaliplatin, irinotecan, fluorouracil, and leucovorin) led to significantly longer survival than gemcitabine among patients with resected PDAC (HR 0.64; 95% CI 0.48 to 0.86; *P* = .003).^[10]^ Thus, modified Folfirinox (mFolfirinox) was changing the standard of care for patients with resected PDAC. However, 2 adjuvant chemotherapy regimens for resected PDAC are the major controversy and ongoing debate area, lacking large prospective randomized controlled trials (RCTs) to offer direct comparison.

Previous network meta-analysis had addressed the effectiveness and safety of adjuvant chemotherapy in patients with resected PDAC. Liao et al^[11]^ found that fluorouracil (HR 0.65, 95% CI 0.49 to 0.84) or gemcitabine (HR 0.59, 95% CI 0.41 to 0.83) was the optimum adjuvant treatment for resected PDAC. However, Xu et al^[12]^ demonstrated S-1 and GX were the most effective adjuvant treatments for resected PDAC in another network meta-analysis. We should pay more attention to the role of adjuvant chemotherapy and differentiate which regimen is better among GX, S-1, and mFolfirinox in patients with resected PDAC.

As mentioned above, for different selected patients, different study design, and variation in the adjuvant therapies, the results that focused on the adjuvant chemotherapy in resected PDAC significantly varied. It is still unclear which adjuvant chemotherapy regimen will be favorable among the all treatments for patients with resected PDAC: monochemotherapy or 2 or 3 drugs combination chemotherapy. The aim of this network meta-analysis is to comprehensively compare different adjuvant chemotherapy regimens to generate evidence and provide suggestions for clinical practice.

## 2 Methods and analysis

### 2.1 Design and registration

A Bayesian analysis of adjuvant chemotherapy following surgery in patients with resected PDAC will be conducted. We will perform this network meta-analysis in accordance with the Preferred Reporting Items for Systematic Reviews and Meta-Analyses Protocols.^[13]^ The Bayesian network meta-analysis was registered with PROSPERO under registration number CRD42019123907 (https://www.crd.york.ac.uk/PROSPERO/). This network meta-analysis did not require ethical approval.

### 2.2 Information sources

We will search PubMed, Embase (Ovid version), Cochrane Library, the American Society of Clinical Oncology, and ClinicalTrials.gov database of titles/abstracts of adjuvant chemotherapy of PDAC until January 19, 2019. The search strategies will be conducted by 2 authors (QCH and XW) who are experienced in the information retrieval. We will manually search related systematic reviews/meta-analyses and bibliographies of included trials to identify additional potential studies.

### 2.3 Search strategy

According to the Population Intervention Comparison Outcomes Study Design framework, the search terms will include the following domains of Medical Subject Heading (MeSH) terms: “Pancreatic Neoplasms” and “Adjuvant chemtherapy”; MeSH and Subheadings were combined with “AND” or “‘OR”.

Two authors will screen the titles/abstracts of related studies independently. Moreover, the eligible or potentially eligible studies will be assessed by reading through the full texts if inclusion criteria are met. In addition, any disagreements will be resolved by having a discussion.

Search strategy of PubMed was as follows:

#1 Pancreas Neoplasms[MeSH Terms]

#2 Neoplasm, Pancreatic[Title/Abstract]

#3 Pancreatic Neoplasm[Title/Abstract]

#4 Pancreas Neoplasms[Title/Abstract]

#5 Neoplasm, Pancreas[Title/Abstract]

#6 Neoplasms, Pancreas[Title/Abstract]

#7 Pancreas Neoplasm [Title/Abstract]

#8 Neoplasms, Pancreatic[Title/Abstract]

#9 Cancer of Pancreas[Title/Abstract]

#10 Pancreas Cancers[Title/Abstract]

#11 Pancreas Cancer[Title/Abstract]

#12 Cancer, Pancreas[Title/Abstract]

#13 Cancers, Pancreas[Title/Abstract]

#14 Pancreatic Cancer[Title/Abstract]

#15 Cancer, Pancreatic[Title/Abstract]

#16 Cancers, Pancreatic[Title/Abstract]

#17 Pancreatic Cancers[Title/Abstract]

#18 Cancer of the Pancreas[Title/Abstract]

#19 #1 OR #2 OR #3 OR #4 OR #5 OR #6 OR #7 OR #8 OR #9 OR #10 OR #11 OR #12 OR #13 OR #14 OR #15 OR #16 OR #17 OR #18

#20 Adjuvant Chemotherapy [MeSH Terms]

#21 Adjuvant Chemotherapy [Title/Abstract]

#22 Drug Therapy, Adjuvant[Title/Abstract]

#23 Adjuvant Drug Therapy[Title/Abstract]

#24 #20 OR #21 OR #22 OR #23

#25 #19 AND #24

### 2.4 Participants

#### 2.4.1 Eligibility criteria

Eligibility criteria are as follows:

1. PDAC.
2. Adjuvant chemotherapy following surgery.
3. RCTs.
4. Report or provide enough information to calculate HRs.
5. The outcomes are OS, disease-free survival (DFS), and grade 3–4 hematological and nonhematological toxicity.
6. There will be no restrictions on status, language, and year of publication.
7. The samples are not subject to any restrictions, such as age, gender, performance status, ethnicity, and country.

#### 2.4.2 Excluding criteria

Excluding criteria are as follows:

1. Adjuvant chemoradiation or targeted therapy following surgery.
2. Periampullary cancer.
3. Posters and abstracts.
4. Cross-sectional, case–control, cohort, or retrospective study designs.

### 2.5 Data collection

We will perform a pilot test between 2 reviewers to evaluate interrater reliability. Then the management of literature search records will be conducted in EndNote X8.

A form will be created for standard data extraction using Microsoft Excel 2007 (Microsoft Corp, Redmond, WA; www.microsoft.com) to collect *point-of-interest data*, such as, name of first author, type of study, recruitment time frame, details of interventions, the sample size, and outcomes (median OS, median DFS, HR, and grade 3–4 hematological and nonhematological toxicity).

The quality and the risk of bias of RCTs will be estimated using the Cochrane Collaboration's tool,^[14]^ which includes 7 specific domains: random sequence generation (selection bias), allocation concealment (selection bias), blinding of participants and personnel (performance bias and detection bias) are tow specific domains, incomplete outcome data (attrition bias), selective outcome reporting (reporting bias), and other bias. The risk of bias for each study will be reported as low risk of bias “+”, high risk of bias “−”, or unclear risk of bias “?”. Two independent authors will complete the assessment of bias risk. The disagreements in assessment will be resolved by having a discussion.

We will use Microsoft Excel 2007 to design a form to summarize data of all the included studies and show their major characteristics and information in this network meta-analysis. The outcomes are median OS, median DFS, and grade 3–4 hematological and nonhematological toxicity. The hematological toxicity is defined as anemia, leukopenia, neutropenia, and thrombocytopenia. Results regarding the OS and DFS are expressed as HRs with 95% CI. Results regarding grade 3–4 hematological and nonhematological toxicity are expressed as odds ratios with 95% CI. *P* < .05 was considered significant. Heterogeneity is assessed with the *I*^2^ statistic. If *I*^2^ < 25%, we consider statistical heterogeneity as low; if 25% ≤ *I*^2^ ≤ 50% as moderate; and if *I*^2^ > 50% as high. If HRs are not reported, we will calculate them via summary statistics with the method described by Tierney et al in 2007.^[15]^ Kaplan-Meier curves will be digitized using Getdata Graph Digitizer 2.26 (www.getdata-graph-digitizer.com). We will perform the traditional pairwise meta-analysis between direct comparisons with Stata13.0 (StataCorp, College Station, TX). The network meta-analysis will be conducted with GeMTC version 1.4.3 (http://drugis.org/software/addis1/gemtc) and WinBUGS version 1.4.3 (MRC Biostatistics Unit, Cambridge, UK). For grade 3–4 hematological and nonhematological toxicity, we will use GeMTC for network meta-analysis. We will use the node-splitting analysis method to evaluate inconsistency. The consistency model will be used when there is no significant inconsistency; otherwise, the inconsistency model will be conducted.^[16]^ The convergence of the model is determined by the potential scale reduction factor of the Brooks–Gelman–Rubin method.^[17]^ Potential scale reduction factor closer to 1 indicates the better convergence. The group with rank probabilities of being the most effective in term of OS, DFS, and safety will be assessed in the network meta-analysis. We will perform a test sequence analysis to evaluate the type 1 error, using TSA 0.9 (Copenhagen TrialUnit, Copenhagen, Denmark).^[18]^

## 3 Discussion

As far as we know, the results of network meta-analysis of RCTs will fill a crucial knowledge gap of effectiveness and safety, especially for GX, S-1, and mFolfirinox as adjuvant chemotherapy for resected PDAC. We hope that the results of the study will help clinicians and patients to select optimum adjuvant treatment in the future.

## Author contributions

**Data curation:** Qiancheng Hu, Xin Wang.

**Formal analysis:** Qiancheng Hu, Xin Wang.

**Methodology:** Qiancheng Hu, Ye Chen.

**Project administration:** Qiancheng Hu, Dan Cao.

**Validation:** Dan Cao.

**Writing – original draft:** Qiancheng Hu, Xiaofen Li, Peng Cao, Dan Cao.

**Writing – review and editing:** Qiancheng Hu, Xiaofen Li, Peng Cao, Dan Cao.
